# Microbial Volatiles: Small Molecules with an Important Role in Intra- and Inter-Kingdom Interactions

**DOI:** 10.3389/fmicb.2017.02484

**Published:** 2017-12-12

**Authors:** Kristin Schulz-Bohm, Lara Martín-Sánchez, Paolina Garbeva

**Affiliations:** Department of Microbial Ecology, Netherlands Institute of Ecology (NIOO-KNAW), Wageningen, Netherlands

**Keywords:** volatile organic compounds, microbial interactions, bacteria, fungi, protists, plant–microbe interactions

## Abstract

During the last decades, research on the function of volatile organic compounds focused primarily on the interactions between plants and insects. However, microorganisms can also release a plethora of volatiles and it appears that microbial volatile organic compounds (mVOCs) can play an important role in intra- and inter-kingdom interactions. So far, most studies are focused on aboveground volatile-mediated interactions and much less information is available about the function of volatiles belowground. This minireview summarizes the current knowledge on the biological functions of mVOCs with the focus on mVOCs-mediated interactions belowground. We pinpointed mVOCs involved in microbe-microbe and microbe–plant interactions, and highlighted the ecological importance of microbial terpenes as a largely underexplored group of mVOCs. We indicated challenges in studying belowground mVOCs-mediated interactions and opportunities for further studies and practical applications.

## Introduction

Many secondary metabolites have been reported to be involved in microbial interactions. One group of secondary metabolites produced by soil and plant-associated microorganisms, but largely unexplored to date, are the volatile organic compounds (VOCs). VOCs are typically small, odorous compounds (<C15) with low molecular mass (<300 Da), high vapor pressure, low boiling point, and a lipophilic moiety. These properties facilitate evaporation and diffusion aboveground and belowground through gas- and water- filled pores in soil and rhizosphere environments (Vespermann et al., 2007; Insam and Seewald, 2010; Effmert et al., 2012). Microbial volatile organic compounds (mVOCs) belong to different chemical classes including alkenes, alcohols, ketones, benzenoids, pyrazines, sulfides, and terpenes (Schulz and Dickschat, 2007; Lemfack et al., 2014, 2017; Kanchiswamy et al., 2015; Schmidt et al., 2015). A recent meta-analysis by Schenkel et al. (2015) provided a comprehensive overview of VOCs derived from soil-borne microbes.

The production of mVOCs in soil is influenced by various factors including the growth stage of the microbes, nutrient availability, temperature, oxygen availability, pH, and soil moisture content (Wheatley, 2002; Insam and Seewald, 2010). Several recent studies reported that the production of certain mVOCs can be induced or suppressed during inter-specific microbial interactions (Garbeva et al., 2014a; Schulz-Bohm et al., 2015; Tyc et al., 2015; Piechulla et al., 2017). mVOCs were often considered to be by-products of primary metabolism, but recent findings revealed that many mVOCs demonstrate biological activity (Schmidt et al., 2015; Tyc et al., 2017a). Furthermore, in bacteria, the production of certain mVOCs is dependent on the GacS/GacA two-component regulatory system (Cheng et al., 2016; Ossowicki et al., 2017). These findings clearly disagree with the opinion that mVOCs are just waste products.

While soluble metabolites are often responsible for short distance interactions, VOCs are considered to be long-distance messengers (Tyc et al., 2017b; Westhoff et al., 2017). There are many types of microbial interactions occurring belowground such as bacteria–bacteria, fungi–fungi, fungi–bacteria, bacteria–protists, fungi–plant, bacteria–plant, and bacteria–fungi–plant interactions. However, most studies addressing belowground VOCs-mediated interactions are focused mainly on the root-emitted volatiles (recently reviewed by Delory et al., 2016).

The knowledge we have gained from research conducted over the last few years reveals that mVOCs can have both beneficial and harmful effects on other organisms (Effmert et al., 2012; Schmidt et al., 2015). mVOCs can provide organisms with rapid and precise ways to recognize neighboring organisms (both friends and foe) and to launch proper responses.

The aim of this review is to summarize the current knowledge concerning the role of mVOCs in intra- and inter-kingdom interactions, to pinpoint mVOCs (e.g., terpenes) involved in microbe–microbe and microbe–plant interactions as well as to indicate challenges in studying belowground mVOCs-mediated interactions and opportunities for further studies and practical applications.

## VOCs in Microbe–Microbe Interaction

### Bacteria–Bacteria

Bacterial VOCs can have direct antagonistic effects against other bacteria. For instance, the sesquiterpene albaflavenone produced by *Streptomyces albidoflavus* revealed activity against *Bacillus subtilis* (Gürtler et al., 1994) and the emission of dimethyl disulphide by two rhizospheric bacteria, *Pseudomonas fluorescens* and *Serratia plymuthica*, showed bacteriostatic effects against two plant bacterial pathogens *Agrobacterium tumefaciens* and *Agrobacterium vitis* (Dandurishvili et al., 2011). *Pseudomonas fluorescens* WR-1 produces volatiles such as benzothiazole and 1-methyl naphthalene with bacteriostatic effects against the tomato pathogen *Ralstonia solanacearum* (Raza et al., 2016a). In fact, many species of *Pseudomonas* and *Bacillus* that are used as biocontrol agents against plant pathogens, have been reported to produce VOCs with antibacterial activity (Raza et al., 2016a,b,c; Xie et al., 2016; Rajer et al., 2017; Tahir et al., 2017a,b). For instance, a recent study revealed that VOCs produced by *Bacillus* spp., including benzaldehyde, 1,2-benzisothiazol-3(2 H)-one and 1,3-butadiene, had strong inhibitory activity against *R. solanacearum*, the causal agent of bacterial wilt disease (Tahir et al., 2017a). The mVOCs altered the transcriptional expression levels of several genes involved in motility and pathogenicity (e.g., global virulence regulator *PhcA*, type III secretion system, and extracellular polysaccharide [EPS] production) and induced systemic resistance by plants, which resulted in a decrease of wilt disease.

Several reports describe the effect of VOCs in bacterial virulence. For instance, 2,3 butanediol and acetoin are required for full virulence in *Pectobacterium carotovorum*. The same compounds can increase the production of virulence factors in *Pseudomonas aeruginosa* (Audrain et al., 2015).

In contrast, VOCs produced by some bacteria can also have positive effects on the growth of other neighboring bacteria in the rhizosphere. For instance, VOCs from *Collimonas pratensis* and *S. plymuthica* are able to induce the growth of *P. fluorescens* Pf0-1 (Garbeva et al., 2014a). These VOCs induced expression of genes involved in motility in *P. fluorescens* Pf0-1 and provoked an increase in the production of secondary metabolites with antibacterial activity against *Bacillus* (Garbeva et al., 2014a). This suggests that *C. pratensis* and *S. plymuthica* may be attracting and promoting the growth of *P. fluorescens* in a collaborative attempt to increase their chances against different bacterial competitors or soil fungal pathogens. Another example of the growth-promoting effect of VOCs was reported recently by Schulz-Bohm et al. (2015) which showed that VOCs released by mixtures of root exudate-consuming bacteria stimulated the activity and growth of distant nutrient-limited bacteria.

In addition to exerting antagonistic effects toward other bacteria, VOCs can also modify the behavior of other bacteria and modulate their resistance to antibiotics. Bacterial volatiles such as ammonia, trimethylamine, hydrogen sulfide, nitric oxide, and 2-amino-acetophenone can alter biofilm formation or dispersal or affect motility of bacteria (Audrain et al., 2015; Raza et al., 2016a). Bacteria often make use of their motility to move to other areas with more resources and/or less competitors. In *Streptomyces venezuelae*, a new mode of development, so-called exploration, has been recently discovered that allows non-motile bacteria to access regions with more nutrients (Jones et al., 2017). *S. venezuelae* is able to produce hydrophilic fast growing non-branching vegetative hyphae, triggered by glucose depletion and a rise in pH, to presumably escape from poor nutrient areas. Interestingly, explorer cells can release signals for long distance communication with other members of the species to induce their exploratory growth. One of these signals is trimethylamine, which works not only as a signal to communicate with distantly located *Streptomyces* and induce exploratory growth but also displays antibacterial activity against *B. subtilis* and *Micrococcus luteus*, probably by rising the pH of the medium (Jones et al., 2017).

### Fungi–Bacteria

Fungal VOCs can play an important role in long distance fungal–bacterial interactions and can lead to different phenotypical responses in the interacting partners. For example, VOCs emitted by *Trichoderma atroviride* increased the expression of a biocontrol gene (*phlA*) in *P. fluorescens* that encodes the biosynthesis of 2,4-diacetylphloroglucinol (Lutz et al., 2004). A few recent studies demonstrated that the growth of some bacterial species can be suppressed by fungal VOCs (Werner et al., 2016) such as the VOCs that exhibit inhibitory effects on *B. cereus* and *B. subtilis* produced by the oyster mushroom *Pleurotus ostreatus* (Pauliuc and Botǎu, 2013).

Recently, Schmidt et al. (2015) screened the phenotypic responses of soil bacterial strains to volatiles emitted by several fungal and oomycetal soil strains under different nutrient conditions during different growth stages. Out of the phenotypical responses tested such as growth alteration, antimicrobial activity, biofilm formation or motility, motility of bacteria (both swimming and swarming) was significantly positively or negatively affected by fungal and oomycetal VOCs. This finding could, therefore, reflect a potential strategy employed by the fungus to attract mutualistic bacteria toward itself and to repel competitors by manipulating their motility through the use of VOCs (Piechulla et al., 2017). Transcriptomics and proteomics analyses of *S. plymuthica* PRI-2C exposed to VOCs emitted by the fungal pathogen *Fusarium culmorum*, showed that *S. plymuthica* PRI-2C responded to the fungal VOCs with changes in gene and protein expression related to motility, signal transduction, energy metabolism, cell envelope biogenesis, and secondary metabolite production (Schmidt et al., 2017). The metabolomic analyses of *S. plymuthica* PRI-2C exposed to the fungal VOCs, the gene cluster comparison, and the heterologous co-expression of a terpene synthase and a methyltransferase revealed the production of the unusual terpene named sodorifen (Kai et al., 2010; Von Reuß et al., 2010) in response to fungal VOCs. These findings support the suggested importance of VOCs (and in particular terpenes) as signaling molecules in fungal–bacterial interactions.

Many soil bacteria can produce VOCs with antifungal effects and thus contribute to the phenomenon known as soil fungistasis where fungal propagules are restricted in their ability to grow or germinate (Garbeva et al., 2011). Recently, Cordovez et al. (2015) revealed that VOCs produced by *Streptomyces* spp. exhibit antifungal properties against *Rhizoctonia solani* and may contribute to plant disease suppressiveness. Ossowicki et al. (2017) showed that VOCs from the tomato rhizosphere isolate *Pseudomonas donghuensis* P482 have strong antifungal and anti-oomycete activity which suggests that the antagonistic capabilities of this strain against plant pathogens are due to their volatile potential (Ossowicki et al., 2017). This effect of bacterial VOCs against oomycetes is not an isolated case and other *Pseudomonas* strains have been reported to have anti-oomycete activities (De Vrieze et al., 2015; Hunziker et al., 2015). In a recent report, VOCs produced by several *Lysobacter* strains growing in a protein-rich medium showed anti-oomycete activity whereas non-antagonistic VOCs were produced by these strains when grown on a sugar-rich medium. This indicates that the production of volatiles is highly dependent on growth conditions and nutrient availability (Lazazzara et al., 2017).

### Fungi–Fungi

The 1-octen-3-ol, one of the most prominent fungal VOC, known as the mushroom smell, is produced by a wide range of filamentous fungi and can function as a development signal among fungi (Miyamoto et al., 2014). The same compound was described to function in *Penicillium paneum* as a self-inhibitor signal in spore germination (Chitarra et al., 2004). As developmental signals during population establishment, certain fungal VOCs act in a concentration-dependent manner to regulate conspecific mycelial growth and spore germination (Nemčovič et al., 2008; Stoppacher et al., 2010).

Fungal VOCs can have inhibitory effects and drive antagonistic interactions among fungi. For example, the endophytic fungi *Muscodor albus* and *Oxyporus latemarginatus* can strongly inhibit the growth of several plant pathogenic fungi, including *Botrytis cinerea* and *Rhizoctonia solani* (Strobel et al., 2001). VOCs emitted by *Trichoderma* spp. have a strong effect against plant pathogenic fungi such as *Fusarium oxysporum, Rhizoctonia solani, Sclerotium rolfsii, Sclerotinia sclerotiorum*, and *Alternaria brassicicola* (Amin et al., 2010). Similarly, VOCs such as 5-hexenoic acid, limonene, octanoic acid and 3,4-2H-dihydropyran produced by the non-pathogenic fungus *F. oxysporum* CanR-46 could inhibit mycelial growth of 14 fungal species including the pathogenic *Verticillium dahlia* (Zhang et al., 2015). Recently, a proteomic study demonstrated that fungal VOCs can interfere with essential metabolic pathways to prevent fungal growth (Fialho et al., 2016).

Some fungal species can detoxify the antifungal compounds produced by their microbial competitors. For example, *Fusarium graminearum* can detoxify the toxic compound 6-pentyl-alpha-pyrone, emitted by *Trichoderma harzianum* (Cooney et al., 2001). Fungal VOCs can be important carbon sources for fungi colonizing carbon-limited environments (Cale et al., 2016). Conversely, for fungi colonizing a more carbon-rich environment, VOCs may act, in a concentration-dependent manner, as semio-chemicals to mediate antagonistic and beneficial interactions between fungi.

### Protists–Bacteria

A very diverse and abundant group of soil microorganisms are protists (Protozoa) (Fierer and Jackson, 2006; Geisen et al., 2015). Due to their grazing activities, protists play an important role in the soil food web and significantly affect carbon allocation and nutrient-cycling in the soil-plant-interphase (Geisen et al., 2016). Most soil protists are known to be key predators of bacteria and can shape bacterial communities by selective feeding (Griffiths et al., 1999; Bonkowski and Brandt, 2002; Rosenberg et al., 2009; Glücksman et al., 2010). Reaching suitable prey is very energy consuming (Jousset, 2012). Thus, sensing their prey over long distances in the porous soil matrix would be very beneficial for protists. A recent study by Schulz-Bohm et al. (2017) revealed that volatile organic compounds can play a key role in long-distance bacterial–protists interactions. By testing various volatile-mediated interactions between phylogenetically different soil bacteria and protists and comparing those with direct trophic interactions, they demonstrated that specific bacterial volatiles can provide early information about suitable prey. In particular, it was shown that terpenes such as β-linalool, β-pinene, germacrene D-4-ol or δ-cadinene produced by *C. pratensis* Ter91 (Song et al., 2015b) can stimulate protist activity and motility suggesting that terpenes can be key components in VOCs-mediated communication between protists and bacteria (Schulz-Bohm et al., 2017). Interestingly, soil protists such as *Dictyostelium discoideum* (Chen et al., 2016) produce volatile terpenes. These terpenes might be involved in defense mechanisms, for example, to repel nematode predators. Similarly, it was shown that soil bacteria can produce specific volatiles to repel protist predators (Kai et al., 2009; Schulz-Bohm et al., 2017).

Besides bacterivorous protists, obligate and facultative mycophageous (fungus grazing) protists are common soil inhabitants (Geisen, 2016). Mycophageous protists feed mostly on yeast and fungal spores while some specialists are able to graze directly on the hyphae of filamentous fungi (Geisen et al., 2016). It is well known that soil fungi such as yeast produce a wide set of volatile compounds involved in various belowground interactions (Effmert et al., 2012; Werner et al., 2016). Thus, although not demonstrated yet, it is plausible that fungal volatiles might play an important role in belowground communication between soil fungi and protists, as well.

## VOCs in Microbe-Plant Interactions

In recent years, evidence supporting the idea that plants respond strongly to mVOCs has grown. Most of the research carried out so far has investigated the impact of microbial VOCs on the model plant *Arabidopsis thaliana*. This has revealed that, without physical contact, microorganisms are able to drastically alter plant root system development, plant physiology, hormonal pathways, and biomass production (Ryu et al., 2004; Blom et al., 2011; Wenke et al., 2012; Bailly et al., 2014; Bitas et al., 2015; Ditengou et al., 2015; Li et al., 2016; Piechulla et al., 2017). mVOCs can also function as a direct source of nutrients for plants (Meldau et al., 2013), induce resistance to pathogens in plants (D’Alessandro et al., 2014; Kottb et al., 2015; Song et al., 2015b; Wintermans et al., 2016), affect plant secondary metabolite production (Santoro et al., 2011), directly inhibit plant pathogens (Kai et al., 2009; Garbeva et al., 2014b; De Vrieze et al., 2015; Kottb et al., 2015) and induce soil fungistasis and suppressiveness (Garbeva et al., 2011; Van Agtmaal et al., 2015). Moreover, one single mVOC can show various functions, such as dimethyl disulfide, which improves plant growth by enhancing the availability of reduced sulfur (Meldau et al., 2013). It also protects tobacco and corn plants against *Botrytis cinerea* and *Cochliobolus heterostrophus* by directly inhibiting pathogens and inducing systemic resistance in plants (Huang C.-J. et al., 2012). Likewise, a characteristic compound of *Trichoderma asperellum*, 6-pentyl-pyrone, can increase plant defense reactions and at the same time decrease *B. cinerea* and *Alternaria alternata* sporulation (Kottb et al., 2015).

Many independent studies revealed that mVOCs emitted by beneficial soil microorganisms can affect plant growth but only few studies focused on how VOCs produced by soil-borne plant pathogens affect plant growth and development. These studies suggest that mVOCs from plant pathogens may modulate the trade-off between plant growth, development and defense. Bitas et al. (2015) showed that VOCs emitted by pathogenic *F. oxysporum* promoted the growth of *A. thaliana* and *Nicotiana tabacum* and affected auxin transport and signaling. VOCs emitted by the pathogen *Alternaria alternaria* enhanced growth, early flowering and photosynthesis rates of *Arabidopsi*s, maize and pepper by affecting the levels of plastidic cytocinin (Sanchez-Lopez et al., 2016). A more recent study showed that the soil-borne pathogen *Rhizoctonia solani* produced an array of mVOCs that promote plant growth, accelerate development, change plant VOCs emission and reduce insect resistance (Cordovez et al., 2017). This must be a successful strategy for the pathogenic fungi since with increased root biomass and stimulation of lateral root formation there is a greater surface area for fungal colonization and infection.

When analyzing mVOCs effects on plant growth, it is important to take into account, that microorganisms can produce high amounts of CO_2_ that can promote plant growth (Kai and Piechulla, 2009; Piechulla et al., 2017). Hence, a good experimental setup with appropriate controls are required to avoid artifacts in the results (Piechulla et al., 2017).

Alternatively, plants are able to mediate the belowground plant–microbe interactions via root-emitted VOCs (Wenke et al., 2010). Root-derived VOCs may serve multiple roles such as carbon sources, defense metabolites and chemo-attractants (Van Dam et al., 2016). Rhizobacteria such as *Pseudomonas fluorescens* and *Alcaligenes xylosoxidans* have been shown to metabolize α- pinene as their sole carbon source (Kleinheinz et al., 1999). Del Giudice et al. (2008) also reported that bacteria associated with the roots of vetiver grass (*Vetiveria zizanioides*) use sesquiterpenes as a carbon source. Undoubtedly, plants and soil microorganisms are engaged via VOCs in long-distance interactions (Van Dam et al., 2016). However, so far, limited knowledge exists concerning the role of plant VOCs in attracting beneficial organisms and how plant-associated microorganisms affect the quantity and quality of plant volatile emission. Only recently, using a glass olfactometer system, the attraction of distant soil bacteria by VOCs emitted by plant roots was revealed (Schulz-Bohm et al., 2017). Olfactometer systems have been used successfully to study aboveground plant–herbivores interactions (Ballhorn and Kautz, 2013) or belowground plant–nematode interactions (Rasmann et al., 2005). However, this is the first case to apply an olfactometer to study plant–microbe interactions. Moreover, the same study revealed that upon fungal infection, the blend of root VOCs changed and specific bacteria with antifungal properties were attracted (Schulz-Bohm et al., 2017).

## mVOCs-Mediated Dialog

Several reports describe the chemical dialog between microbes, plants, and other organisms by the exchange of soluble compounds (Badri et al., 2009; Lira et al., 2015; Song et al., 2015a; Liu et al., 2016). Most of the studies reporting mVOCs-mediated communication belowground focus on the uni-directional responses and only a few studies reported on bi-directional mVOCs-mediated interactions. For instance, the importance of mVOCs in the dialog between the fungal plant pathogen *Verticillium longisporum* and its bacterial antagonist *Paenibacillus polymyxa* was recently revealed in both *in vitro* and *in planta* experiments (Rybakova et al., 2017). Both microorganisms responded to one another’s VOCs and this specific mVOCs-mediated interaction resulted in the inhibition of cellular metabolism and growth reduction of the fungal pathogen.

A VOCs-mediated dialog between bacteria and fungi was also reported by Spraker et al. (2014) where VOCs of the fungal plant pathogen *Aspergillus flavus* reduced the production of the major virulence factor EPS of the bacterial plant pathogen *R. solanacearum.* In parallel, *A. flavus* responded to VOCs of *R. solanacearum* by reducing conidia production and increasing aflatoxin production.

## Conclusion and Outlook

**FIGURE 1:**
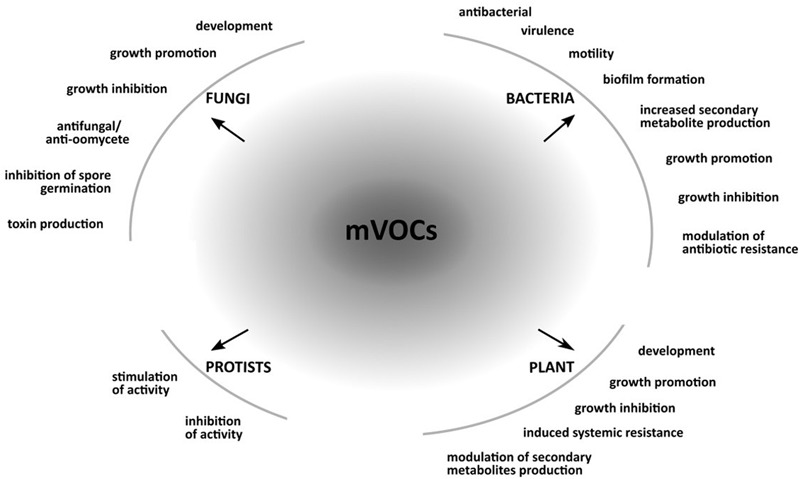
Responses in bacteria, fungi, protists and plants caused by microbial volatile organic compounds (mVOCs). The figure shows examples of responses caused my mVOCs in bacteria, fungi, protists, and plants.

Over the last decades, our understanding of the chemical complexity of mVOCs produced by many different soil microorganisms has grown. It is clear that these small and odorous molecules can modify the behavior and promote or inhibit growth of neighboring organisms (**Figure 1**).

Most existing studies on mVOCs are focused on describing the uni-directional effect of mVOCs produced by a single organism and the responses of the organisms perceiving them without considering mVOCs-mediated dialog and the bi-directional responses to one another. Furthermore, microbial interactions taking place belowground are far more complex than single one-to-one interactions and involve more organisms, which can significantly affect mVOCs emission. For example, fungal-associated bacteria have been shown to affect the production of VOCs in fungi (Schulz-Bohm et al., 2015; Splivallo et al., 2015) and in addition, they can affect the fungal plant-pathogenicity and repress the expression of fungal virulence genes (Minerdi et al., 2009). Therefore, a holistic approach considering the effect of mVOCs on belowground soil community is needed. For instance, using a metagenomics approach Yuan et al. (2017) revealed that mVOCs could alter the composition of soil bacterial and fungal communities and significantly increased the relative abundance of *Proteobacteria, Bacteroidetes, Firmicutes*, and *Ascomycota*. Furthermore, mVOCs influenced genes involved in important soil functions such as N-fixation (*nif*H), nitrification (*amo*A), denitrification (*nir*S) and antibiotic production (NRPS) (Yuan et al., 2017).

From the current scientific literature, it is clear that the most studied belowground mVOCs-mediated interactions are the interactions between bacteria, fungi and plants (**Figure 1**). There is a lack of knowledge relating to the emission of VOCs by protists, archaea or other rhizosphere organisms, such as nematodes or earthworms, indicating that these groups are currently understudied with regards to this aspect.

**FIGURE 2:**
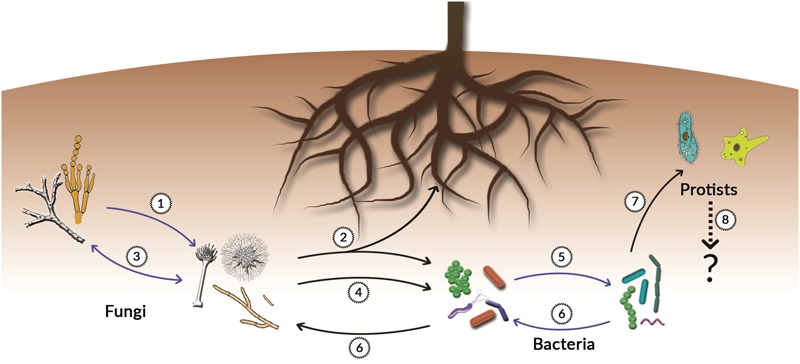
Terpenes-mediated belowground interactions. The figure shows examples of interactions between different organisms in the rhizosphere that are mediated by microbial terpenes. Blue arrows indicate intra-kingdom interactions while black arrows indicate inter-kingdom interactions. The numbers in the figure correspond with the numbers in **Table 1**.

Several VOCs are commonly produced and emitted by both plant roots, fungi, bacteria and protists and it is possible that these compounds act as a ‘lingua franca’ for intra- and inter-kingdom communication between these organisms. Let us take as an example only one chemical class, the terpenes. Terpenes are the largest and most diverse class of metabolites known to date. They are best known to humans as plants metabolites. However, recent studies revealed that terpenes can be produced by all kingdoms of life including prokaryotes (Takamatsu et al., 2011; Yamada et al., 2012, 2015; Song et al., 2015b; Chen et al., 2016). Recently, Yamada et al. (2015) described a powerful bioinformatics method based on the use of Hidden Markov Models (HMMs) and Protein Families Database (PFAM) search that has allowed the discovery of terpene synthases of bacterial origin and showed that phylogenetically different bacteria can be a rich source of terpenes. Both the number, the wide distribution, and the structural diversity of terpenes provide enormous potential for mediating significant chemical interactions and communication belowground. Examples of terpene-mediated microbial interactions are presented in **Figure 2** and **Table 1**, indicating the ecological importance of terpenes in interactions between soil micro- and macro-organisms, including plant roots.

**Table 1:** Examples of terpenes involved in belowground microbial interactions.

Origin	Nr	Compound	Biological activity	Reference
Fungal	1	α –Humulene	Antimicrobial (antifungal)	Minerdi et al., 2009
	2	β -Caryophyllene	Antimicrobial (antibacterial)	Minerdi et al., 2011; Huang M. et al., 2012
			Plant growth promotion	
	3	Farnesol	Infochemical	Hornby et al., 2001; Martins et al., 2007
	4	β-Phellandrene	Affects motility	Schmidt et al., 2017
Bacterial	5	Albaflavenone	Antimicrobial (antibacterial)	Gürtler et al., 1994
	6	β-Pinene	Antimicrobial (antifungal, antibacterial)	Garbeva et al., 2014b; Song et al., 2015b
	7	Volatile terpenes from *Collimonas*	Stimulation of protists activity	Schulz-Bohm et al., 2017
Protist	8	(E,E)- α-farnesene β-barbatene	Unknown	Chen et al., 2016

Despite the rapid increasing numbers of studies showing the importance of mVOCs in the long-distance belowground chemical interactions, we still do not know exactly how VOCs are recognized and perceived. VOCs receptors or other perception mechanisms have not been identified in any of the described cases. The big challenge is to determine whether VOCs are internalized and transduced by receptor-mediated processes, whether they interact with the cell membrane to initiate signal transduction cascades or whether they are simply taken up by the cell and metabolized (Widhalm et al., 2015; Adebesin et al., 2017; Tissier et al., 2017). For plants, the current view is that due to their lipophilic nature, VOCs such as mono- and sesquiterpenes may interfere with membrane structures, thereby causing depolarization of the membranes and triggering Ca^2+^-signaling in plants (Maffei et al., 2001; Heil and Land, 2014). For further deciphering of mVOC-mediated microbe-microbe interactions, the mVOCs microbial perception mechanism needs to be elucidated. The application of methods for screening of mutant strains may be useful for that purpose, to identify microbial genes and proteins that are required for VOCs perception.

Another big challenge is to determine what concentrations of mVOCs are produced in soil and at what distances these mVOCs are eliciting a biological response in other organisms. There is the possibility that, similar to the roles of antibiotics in nature (Davies et al., 2006; Yim et al., 2006; Romero et al., 2011), mVOCs could have concentration-dependent function either as weapons in intercellular chemical warfare or as signaling compounds when they are present in low concentrations.

Concerning the implementations of mVOCs, our knowledge on the potential use of those compounds in large-scale agriculture and horticulture is still limited. In agriculture systems, mVOCs have to be applied under open-field conditions, which are very different from the *in vitro* conditions currently used in most studies. There are very few studies assessing the effects of mVOCs application under open conditions and they have been summarized in a recent review from Chung et al. (2016). Since it was discovered that the 2,3-butanediol elicited plant growth and induced systemic resistance (Ryu et al., 2003, 2004), several studies have applied this compound or the producing strains to the soil of open fields to test its effects under agricultural conditions and have revealed promising results (Velivelli et al., 2015). Dimethyl disulfide, frequently emitted by many bacteria, is another compound used in recent years in the novel soil fumigant PALADIN^®^ that targets nematodes and soil-borne pathogens. However, the research concerning the application of other mVOCs in agriculture is still in its infancy. We now live in a time in which the old methods of using chemicals to protect crops need to be replaced with and, in some cases, complemented by green solutions. The traditional harmful synthetic fungicides currently used could be replaced with the so far under-explored and unique mVOCs for which significant proof of plant growth promoting effects and plant protection ability already exists. In spite of the obvious potential of mVOCs in agriculture, the field suffers from the common ‘translational gap’ because of a lack of studies evaluating other unexpected effects of those bioactive molecules on non-target beneficial soil organisms.

## Author Contributions

All authors contributed and approved the manuscript.

## Conflict of Interest Statement

The authors declare that the research was conducted in the absence of any commercial or financial relationships that could be construed as a potential conflict of interest.
